# Comment on Ramos-Rincon et al. Palliative Sedation in COVID-19 End-of-Life Care. Retrospective Cohort Study. *Medicina* 2021, *57*, 873

**DOI:** 10.3390/medicina58010082

**Published:** 2022-01-06

**Authors:** Robert Geoffrey Twycross, Aaron Kee Yee Wong, Bella Vivat

**Affiliations:** 1 Palliative Medicine, University of Oxford, Oxford 0X1 2JD, UK; 2 Department of Palliative Care, The Royal Melbourne Hospital, 300 Gratton Street, Parkville, VIC 3050, Australia; aaron.wong@petermac.org; 3 Marie Curie Palliative Care Research Department, Division of Psychiatry, University College London, London W1T 7NF, UK; b.vivat@ucl.ac.uk

We read with interest the article by Ramos-Rincon and colleagues about patients with COVID-19 dying in acute medical wards in a Spanish University hospital [1]. However, this article illustrates how “palliative sedation” is commonly used in conflicting ways [2,3]. The Introduction defines it as “inducing a state of decreased or absent awareness (unconsciousness) to relieve the burden of otherwise intractable suffering until death”, i.e., sedation to the depth necessary to alleviate the patient’s suffering. This phrasing aligns with both the European Association of Palliative Care (EAPC) recommended framework for the use of sedation in palliative care [4] and the Guía de sédacion paliativa of the Organización Médica Colegial de España and Sociedad Española de Cuidados Paliativos [5].

“Palliative sedation” thus implies an intentional switch to using sedative medication to reduce a person’s level of consciousness, rather than for incremental symptom management. In practice, this will sometimes mean only light sedation (drowsy but responding to voice), and at other times, continuous deep sedation (responding only to physical stimulus or totally unresponsive). Therefore, without qualification, “palliative sedation” does not imply any particular *depth*; it can mean any level of sedation until death, including, more narrowly, continuous deep sedation (CDS).

However, in the Materials and Methods section, the authors unambiguously define “palliative sedation” solely as CDS. Thus, it appears that the article is not about the whole of “palliative sedation”, but specifically CDS. This narrower focus does not sit well with the statements in the Introduction that the WHO definition of palliative care implies that palliative care necessarily entails palliative sedation, and that “the use of palliative sedation is an indicator of the quality of end-of-life care”.

These two statements are debatable in general, specifically with regard to CDS. The second is particularly surprising because it is supported by reference to an article which nowhere mentions palliative sedation, but rather discusses the use of midazolam and morphine for symptom management in patients in an Australian tertiary care hospital dying from COVID-19 [6]. It is also ambiguous: does the use of palliative sedation indicate that the care is of high quality or low quality? The context suggests that the intended meaning is “high quality”, but we question whether this can be claimed without elaboration. Although the incidence of CDS reported by specialist palliative care services worldwide ranges up to about 15% [2], in three countries (Belgium, Colombia and Japan), much lower incidences have been reported (2.4%, 2% and 1.4% respectively) [7,8,9]. It could be that the need for CDS is higher, even much higher, for patients dying from COVID-19 than in pre-pandemic palliative care populations, but it is important to discover whether this is so.

The study’s inclusion of patients whose death was not directly related to COVID-19 and who died from other conditions (about 25% of the total; Table 1) is a potential confounding factor. Further, the drugs and doses presented in Table 2 look very similar to those used in routine symptom management. The drugs listed in the sedative infusions include hyoscine butylbromide—used to reduce “death rattle” but totally non-sedative—so we wonder whether the authors are just reporting the incidence of the use of morphine and other drugs in these patients. Information on the doses these patients were receiving before moving to “palliative sedation” would help in deciding whether any increase in an infusion could, perhaps, be considered as the next step in symptom management or a deliberate shift to an intentional suppression of consciousness.

The most common symptoms treated by CDS in this study were dyspnea at rest, pain and agitation/delirium. The median doses of morphine and midazolam used were 40 mg and 15 mg, respectively (presumably per 24 h, although not stated). For the same three common reasons in the Australian study (which, as already pointed out, did not investigate palliative sedation), the median final 24 h doses of morphine and midazolam were 45mg and 12.5 mg, respectively [6]. These doses are almost identical in both studies and are in keeping with those typically used for symptom management, as are the psychotropic drug doses in the Spanish study, namely, haloperidol 10 mg and levomepromazine 75 mg.

The median duration for “palliative sedation” of about 22 h (IQR 10–41) reported in this study suggests that many of the patients included were very close to death. For most people, a reducing level of consciousness leading to coma is part of the natural dying process: progressive organ failure results in the accumulation of toxic metabolites, which leads to depression of both the cerebral cortex and brainstem. Sedatives will tend to accelerate and potentiate both a reduction in level of consciousness and cardiorespiratory failure. The relative contributions of sedative medication and of natural dying to the depth of reduced consciousness are difficult to determine. However, we suggest that in most people, reduced consciousness at the end of life is probably *not* palliative sedation or deliberate CDS, but is largely secondary, i.e., the co-incidental result of enhanced symptom management. Precision is therefore essential when discussing the use of sedatives at the end of life, particularly when using the term “palliative sedation”.
